# The Potential Use of Mesenchymal Stem Cells and Their Derived Exosomes as Immunomodulatory Agents for COVID-19 Patients

**DOI:** 10.1155/2020/8835986

**Published:** 2020-09-24

**Authors:** Faisal A. Alzahrani, Islam M. Saadeldin, Abrar Ahmad, Dipak Kumar, Esam I. Azhar, Arif Jamal Siddiqui, Bassem Kurdi, Abdulrahim Sajini, Abdulmajeed F. Alrefaei, Sadaf Jahan

**Affiliations:** ^1^Department of Biochemistry, Faculty of Science, Embryonic Stem Cell Unit, King Fahad Center for Medical Research, King Abdulaziz University, Jeddah, Saudi Arabia; ^2^Department of Physiology, Faculty of Veterinary Medicine, Zagazig University, Zagazig 44519, Egypt; ^3^Department of Animal Production College of Food and Agriculture Science, King Saud University, Riyadh 11451, Saudi Arabia; ^4^Zoology Department, KKM College, Munger University, Jamui, India; ^5^Department of Medical Laboratories, College of Applied Medical Sciences, King Abdulaziz University, Jeddah, Saudi Arabia; ^6^Department of Biology, College of Science, University of Hail, Hail, Saudi Arabia; ^7^Department of Pediatrics, Faculty of Medicine, King Abdulaziz University, Jeddah, Saudi Arabia; ^8^Department of Biomedical Engineering, Khalifa University of Science and Technology, Abu Dhabi, UAE; ^9^Jamoum University College, Department of Biology, University of Umm Al-Qura, Saudi Arabia; ^10^College of Applied Medical Science, Majmaah University, Al Majmaah, Saudi Arabia

## Abstract

A novel severe acute respiratory syndrome coronavirus (SARS-CoV-2) causing lethal acute respiratory disease emerged in December 2019. The World Health Organization named this disease “COVID-19” and declared it a pandemic on March 11, 2020. Many studies have shown that mesenchymal stem cells (MSCs) and their exosomes (MSCs-Exo), which are isolated from allogenic bone marrow stem cells, significantly lower the risk of alveolar inflammation and other pathological conditions associated with distinct lung injuries. For example, in acute respiratory distress syndrome (ARDS) and pneumonia patients, MSCs-Exo and MSCs provide similar healing properties and some clinical trials have used cell-based inhalation therapy which show great promise. MSCs and MSCs-Exo have shown potential in clinical trials as a therapeutic tool for severely affected COVID-19 patients when compared to other cell-based therapies, which may face challenges like the cells' sticking to the respiratory tract epithelia during administration. However, the use of MSCs or MSCs-Exo for treating COVID-19 should strictly adhere to the appropriate manufacturing practices, quality control measurements, preclinical safety and efficacy data, and the proper ethical regulations. This review highlights the available clinical trials that support the therapeutic potential of MSCs or MSCs-Exo in severely affected COVID-19 patients.

## 1. Introduction

A lethal acute respiratory tract disease caused by a novel severe acute respiratory syndrome (SARS) coronavirus emerged at the end of 2019 in Wuhan, China [1–3]. The first outbreaks in China 13.8% suffered severe disease and 6.1% required critical care [4]. Since that outbreak, the World Health Organization (WHO) named the disease Coronovairus Disease “COVID-19” and declared it a pandemic on March 11, 2020 [5]. It is caused by an RNA virus (ssRNA) 50–200 nm in diameter that is composed of four structural proteins: nucleocapsid protein, spike protein, envelope protein, and membrane protein [1].

As COVID-19 cases emerged, the pertaining symptoms were associated with severe respiratory tract infections and inflammations. At first, the infections were thought to be part of the normal, seasonal flu. However, after many failed attempts to control the infectious virus, it was identified as a different virus with similar symptoms to other respiratory viral diseases. Patients probably were first infected with the virus through a wholesale market of seafood and other nonvegetarian food items; the first patients worked at the market or made purchases there regularly. As more cases were identified, the market was closed with immediate effect, and all required steps were adopted to avoid further spreading of infections due to the highly contagious nature of the virus [4].

The causative virus was found to have a 5% genetic association with SARS as part of a subset of beta coronaviruses [6]. The WHO identified the virus as severe acute respiratory syndrome coronavirus-2 (SARS-CoV-2) and recommended that the disease resulting in the current outbreak should be explained as “2019-nCoV acute respiratory disease” (2019 novel coronavirus acute respiratory disease). The nomenclature for the virus was confirmed by the International Committee on Taxonomy of Viruses (ICOTV) as SARS-COV-2 [7].

Rapid replication of SARS-CoV-2 is believed to occur after the onset of infection and severe inflammatory responses due to cytokine storms have been observed. This subsequent inflammatory response damages alveolar epithelia and capillary endothelial cells, resulting in interstitial and alveolar edema and impaired pulmonary functions. Such damage leads to acute hypoxic respiratory failure and results in acute respiratory distress syndrome (ARDS). People older than 50 years are at a high risk for COVID-19-induced pneumonia, and the WHO has estimated the mortality rate of SARS-CoV-2 to be ~3.7% [8].

Due to its appearance under an electron microscope, which is like a solar corona, the SARS-CoV-2 family was named *Coronaviridae*. The subfamily *Orthocoronavirinae* is zoonotic and is further categorized into the following genera: alpha, beta, gamma, and delta coronaviruses. Coronaviruses are varied and have single-strand (ss), positive-sense RNA (+RNA) [7]. Enzyme lactate dehydrogenase levels and neutrophil counts are used as disease identification markers for SARS viruses [9]. Evidence suggests that bats and birds are the primary hosts for these coronaviruses, and many studies have suggested that coronaviruses can infect bats, birds, cats, dogs, lions, pigs, mice, horses, and whales, as well as humans. Genomic and serologic data have confirmed that camels and bats can act as intermediate hosts for humans for Middle East Respiratory Syndrome Coronavirus (MERS-CoV) and SARS-CoV, respectively.

Phylogenetic studies of the complete RNA-dependent RNA polymerase (RdRp) gene have shown that SARS-CoV-2 is different from SARS-CoV; therefore, SARS-CoV-2 has been identified in the subgenus *Sarbecovirus* [7]. The genomic studies of SARS-CoV-2 have revealed its similarity to bat-derived coronavirus strains, such as bat-SL-CoVZC45 and bat-SLCoVZXC21, the virus that caused the SARS outbreak in 2003 [7]. Evidence has also revealed that SARS-CoV-2 survives on distinct surfaces. For example, the virus can persist for 3 hours in aerosol and up to 72 hours on stainless steel, plastic, cardboard, and copper surfaces [10].

One of the guidelines recently released by the National Health and Medical Commission indicated that SARS-CoV-2 severe cases generally impart severe pneumonia accompanied by difficulty in breathing after one week of illness [11, 12]. Severe cases quickly progress to ARDS, septic shock, and multiple organ dysfunction syndrome (MODS), which are challenging to manage medically [13]. As mentioned previously, COVID-19 causes a severe secretion of cytokines in the lung region, thereby damaging the alveolar epithelia and capillary endothelial cells. The immunomodulatory effect of the therapeutic agents can reverse this reaction and thus protect the lungs from severe damage [14].

The containment, management, and treatment of the disease have challenged both clinicians and researchers in different fields of biomedicine. As of the finalization of this manuscript, no positive or promising treatment against SARS-CoV-2 has been found, although many streamlined therapies are on the way. However, the side effects of some pharmaceutical drugs that have been used to try to control the disease have been observed and drugs are effective in some individuals, while they fail in others. Convalescent plasma therapy also showed some promise [15–17]. Researchers are also working on a vaccine against COVID-19 but still have a long way to go. Therefore, based on the research and generated data, immediate therapy that does not negatively impact the health of the patients is still needed to overcome the crisis.

Stem cell research and therapy have given hope to both researchers and clinicians. Previous studies have shown that stem cell therapy is a promising treatment for numerous diseases and conditions, such as neurodegeneration, diabetes, and cancer. [18, 19]. Recently, mesenchymal stem cells (MSCs) have been introduced as a potential therapeutic approach for treating SARS-CoV-2 [19]. MSCs suppress viral infections by releasing specific cytokines; these features are intrinsically present while the MSCs reside in their niche before being isolated from the source tissue [20]. Therefore, MSCs and their exosomes (MSCs-Exo) are expected to survive even when transplanted into a patient with a confirmed SARS-CoV-2 infection (NCT04276987). Due to the ambiguity of MSC therapy in treating SARS-CoV-2, the reported clinical trials are being reviewed to present the information to researchers of the stem cell-based therapy. This review focuses on approaches to improve patients' immunological response against SARS-CoV-2 infection using MSCs and/or MSCs-Exo therapy.

## 2. Covid-19 Diagnosis and Pathogenesis

Initially, SARS-CoV-2 infection presents mild symptoms that are similar to diseases caused by other respiratory viruses [21]. The timeline between exposure and appearance of the first symptoms ranges from 1 to 14 days [4], and epidemiological studies have revealed that SARS-CoV-2 may be transmitted during the pre-symptomatic incubation period [22]. Indeed, virologic studies using reverse transcriptase-polymerase chain reaction (RT-PCR) have detected large quantities of the SARS-CoV-2 viral RNA among persons with asymptomatic and pre-symptomatic SARS-CoV-2 infections [23]. The risk of transmission is not yet clear due to the uncertain degree of SARS-CoV-2 viral RNA shedding. However, it is thought to be greatest in symptomatic patients since viral shedding is at its peak at symptom onset and gradually declines over time (up to several weeks) [22–24].

The symptoms of SARS-CoV-2 infection may involve a dry cough, sore throat, tiredness, high fever, anorexia, myalgia, and nasal congestion among others [25]. These symptoms begin increasing gradually, leading to difficulty in breathing and need for hospitalization as they progress. Less than 10% of symptomatic patients have reported headaches, confusion, hemoptysis, rhinorrhea, sore throat, vomiting, and diarrhea [26]. Some persons with SARS-CoV-2 may experience gastrointestinal symptoms, such as diarrhea and nausea, before the onset of fever and lower respiratory tract problems [27]. About 80% of symptomatic patients do not require medical assistance or hospitalization. The rate of mortality reported among patients admitted to the ICU has varied, ranging from 39 to 72%. Survivors have a median hospitalization period of 10–13 days [4].

Two other lethal coronaviruses, SARS-CoV and MERS-CoV, have been found to induce an excessive and unusual immune response of the host cells similar to SARS-CoV-2 [28]. However, unlike SARS-CoV-2, infections caused by these viruses are always accompanied by cytokine storms (an encroachment of the immune system cells and their activating compounds, such as cytokines) that subsequently result in ARDS, thereby producing multiple organ failure and/or death [8]. Even in patients treated for cytokine storms in the ICU, continued inflammation leads to severe pulmonary fibrosis, causing lung dysfunction and substantially reducing the quality of life [8]. The pathogenesis of SARS-CoV-2 is less clear, and many avenues for new therapeutic strategies are being explored and unfortunately, nothing is reliable at this time.

## 3. Immunomodulation by Mesenchymal Stem Cells and/or Their Exosomes

Therapeutic applications of stem cells for a variety of disorders have been explored. As a result, the number of clinical trials conducted with MSCs has increased exponentially over the past few years. MSCs are the most efficient and postindigenous stem cells as they are self-renewable and able to differentiate into multiple genealogies [29, 30]. These stem cells possess atypical characteristics, such as easy isolation and harvesting procedures, pliability, and intrinsic movement toward an injured area (i.e., the process of homing) [31–33]. MSCs have shown antiapoptotic and anti-inflammatory actions in the administrated tissues, and through paracrine secretions, MSCs are responsible for immunomodulatory effects [34, 35]. Furthermore, they are capable of activating other resident stem cells to be utilized in the healing process and can stimulate neo-angiogenesis, tissue repair, and cell survival in surrounding tissues [36] by facilitating tissue regeneration through mechanisms involving their inherent self-renewal and multiple differentiations. These capabilities have rendered MSCs biologically significant and clinically useful for research. Notably, MSCs are free from any ethical issues and constraints enabling their universal application. MSC therapy has been effectively utilized to cure some disorders, including degenerative, inflammatory, and metabolic diseases, and it has been used to repair and regenerate damaged or lost tissues [30, 31, 37, 38]. Moreover, the therapy has been found to be applicable for treating cancer [39] as well as neurodegenerative disorders [40].

Every stem cell, including MSCs, has a distinctive and intrinsic homing property that moves it toward the site of inflammation in the body. Various studies have indicated that stem cells can repair damaged or diseased tissues, ease inflammation, and modulate the immune system so that the patients' quality of life is improved. These stem cells influence tissue repair via paracrine activities or by direct cell-to-cell contacts. In the case of injury, MSCs migrate toward the injured area, where they generate numerous cells and play an important role in healing via high multiplication, differentiation immunomodulation, and neovascularization induction [36, 37, 41, 42]. Based on their use in successful transplantation therapy, the stem cells are considered a single arrow that can hit multiple targets (i.e., various diseases), resulting in novel therapeutic outcomes [29].

Exosomes are released from all kinds of cells, including stem cells [43]. They have shown molecular similarity with their mother cells; therefore, it has been hypothesized that MSCs-Exo can be administered to the affected area for neovascularization and tissue repair [41]. Exosomes are membrane-enclosed extracellular vesicles (EVs) that have a diameter ranging from 30 to 100 nm. Studies of exosomes have reported that they are stable at low storage temperatures for long periods [44], and this survival rate and stability make them superior to the parent cell [45, 46]. Furthermore, when compared to cellular therapy, exosomes and EVs provide an appealing, prudent, and promising therapeutic strategy because they lack nuclei, which frees them from the risk of tumor formation and any kind of mutation [47]. Despite lacking a nucleus, exosomes have all the essential growth factors and biological signals that help restore damaged tissues [48, 49]. Indeed, analysis of exosomes' genomics and proteomics data found that the quality of their mRNA, miRNA, tRNA, and protein is first rate [50–53].

Experimental studies have shown the efficacy of MSCs and MSCs-Exo in treating pathological conditions, including reducing lung inflammation. For example, when MSCs and MSCs-Exo were injected into pneumonia patients, the pertaining trials exhibited nearly similar therapeutic effects [54, 55]. On the other hand, cell-based inhalation therapy for treating lung infections has also progressed to clinical trials [56, 57]. Additionally, some studies have suggested the immunomodulatory effects of MSCs and MSCs-Exo. An animal model of bronchopulmonary dysplasia showed that exosomes derived from both the umbilical cord and bone marrow reduced inflammation, fibrosis, pulmonary hypertension, and pulmonary vascular modeling, thereby improving lung function [38]. The mechanism involved in these improvements may have been a modulation of the phenotype of macrophages with an increase in the number of immunosuppressive M2 macrophages [38]. Furthermore, peripheral blood mononuclear cells (PBMC) were isolated from asthmatic patients and treated with bone marrow MSCs-Exo in *in vitro* conditions; this treatment increased the expression of interleukin-10 (IL-10) and transformed growth factor beta 1 (TGF-*β*1), thus enhancing the function of immunosuppressive regulatory T cells [58]. Moreover, exosomes taken from adipose tissue-derived MSCs were found to reduce atopic dermatitis in an *in vivo* mouse model; this effect was mediated through a reduction in the levels of inflammatory cytokines, eosinophils, infiltrated mast cells, IgE, and CD86+ and CD206+ cells [59].

In a skin-defect mouse model, exosomes from umbilical cord MSCs were found to reduce scar formation and accumulation of myofibroblasts [60], while a rat skin burn model showed that exosomes from umbilical cord MSCs both enhanced the ability of skin wounds to reepithelialize and promoted the ability of skin cells to proliferate and survive [61]. Another study using the rat skin burn model found that umbilical cord MSCs-Exo reduced burn-induced inflammation; this was attributed to the expression of exosomal miRNA-181c [62]. Interestingly, in an *in vivo* model of skeletal muscle injury, bone marrow MSCs-Exo were found to enhance the regeneration of skeletal muscle, which was attributed partly to miRNAs contained in the exosomes [63]. Additionally, pathological damage due to inflammation in a chronic graft-versus-host-disease mouse model was found to be ameliorated by treatment with bone marrow MSCs-Exo through a reduction in the activation and infiltration of CD4+ T cells, suppression of T helper 17 cells, reduction in inflammatory cytokines, and increase in the levels of regulatory T cells [64].

Various studies have estimated that MSCs play a crucial role in tissue regeneration and immunomodulation through their paracrine activity. Moreover, MSCs facilitate antiapoptotic activity, impeding the fibrosis of tissues. One study showed the ability of MSCs to reduce microbial-induced lung injuries in an *in vivo* mice model; they imparted significant contribution against H9N2 and H5N1 viruses in *in vivo* mouse models by reducing the hypersecretion of cytokines into the lungs [65].

The International Society for Cellular and Gene Therapies (ISCT) and the International Society for Extracellular Vesicles (ISEV) do not currently endorse the use of EVs or exosomes for any purpose in COVID-19 due to some valid points before administration to the COVID-19 patients. Although the ISCT and ISEV encourage research and trials of MSCs-Exo, they suggest strict handling and precautionary measures while using MSCs-Exo-related therapies to avoid failure and risk to the subject's health.

The source of the MSCs-Exo is also important as MSCs are a heterogeneous cell entity that can be obtained from different tissues. Even if they are obtained from identical tissues, they may display clone-specific functional differences [66]. Side-by-side comparison of four MSCs-Exo preparations harvested from the conditioned media of different donor-derived bone marrow MSCs showed significant variations in cytokine content (Kordelas et al. 2014). Additionally, the correlation with MSCs' therapeutic potency is poorly understood, and researchers must identify the exact mechanistic action [67].

Like some other viruses, the novel coronavirus enters the host cell through the angiotensin-converting enzyme 2 (ACE2) receptor on the host cell's surface [68]. ACE2 receptors are abundantly present in human blood vessels and act as a cardioregulator. In the lungs, these receptors are present on the alveolar type II cells (AT2). This is the reason that coronavirus mostly attacks the cells of the capillary-permeated lungs [69]. Additionally, the virus can affect any other organ where ACE2 receptors are present, such as the kidneys. Indeed, multiorgan failure in severe cases can even result in patients' deaths.

**Figure 1:**
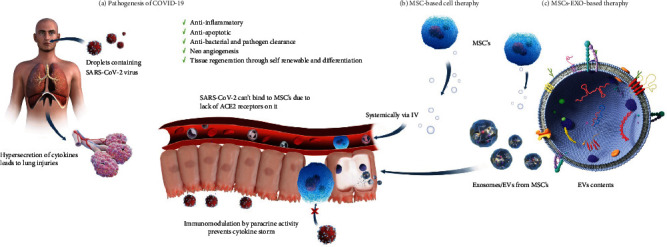
Pathogenesis of COVID-19 and stem-cell-based therapy. (**a).** SARS-CoV-2 enters into the human body via droplets from infected patients. In human cells, SARS-CoV-2 binds with the ACE2 receptors present on host cells and initiates a cytokine storm. This storm results in severe lung injury. (**b).** MSCs. (**c).** MSCs-Exo or EVs are considered a possible future treatment due to many of their properties, such as a lack of ACE2 receptors (which prevents a cytokine storm) and immune modulation and restoration of damaged cells due to their essential growth factors and metabolites, see Supplemental Video 1 for the proposed mechanism of MSCs-Exo in treating COVID-19 symptoms.

In this case, MSCs can be deployed to ameliorate SARS-CoV-2-induced hyperinflammation and pathology through their anti-inflammatory activity. As ACE2 receptors are absent on MSCs, MSCs cannot be targeted by COVID-19. Thus, MSCs can a reliable treatment for overcoming lung injuries and can be involved in the antiviral pathway [70]. Additionally, MSCs can interact with most of the cells of the immune system, such as B cells, T cells, neutrophils, natural killer (NK) cells, dendritic cells (DCs), and macrophages, and thereby moderate these cells' response to pathogens [58, 66]. Moreover, MSCs are stimulated by inflammatory cytokines only when inflammation levels are uncontrollably high. Furthermore, MSC-released cytokines can prohibit neutrophil dissemination and improve macrophage differentiation [71]. Additionally, they can release EVs containing microRNAs, mRNAs, DNA, proteins, and other metabolites that can specifically be delivered into host lung cells, thereby promoting the regeneration and restoration of lung structures and functions [72]. With such astonishing properties, MSCs-Exo could prove to be a promising therapy for COVID-19. The pathogenesis and rescue therapy of MSCs are presented in Figure 1.

## 4. Recent Studies and Clinical Trials Using Stem Cells against COVID-19-Induced Pathogenesis

Cell-based clinical trials using stem cells—especially MSCs and MSCs-Exo from variable sources like adipose tissue, umbilical cord blood (UCB), Wharton's jelly, and bone marrow—in the treatment of ARDS are undergoing. Indeed, some of the ongoing clinical trials have yet to submit their final reports (see Table 1). The safety of MSC application is being documented, and most reports show low mortality and morbidity rates in COVID-19 patients [73]. One study reported the effectiveness of MSC therapeutic strategies against threatening COVID-19-induced immune system reactions [74]. Moreover, Leng et al. [75] used MSCs to treat seven cases (two common, four severe, and one critically severe case). They showed improvement in the pulmonary functional outcome of all seven patients in day 2 of MSC injection without any adverse effects. Furthermore, Sengupta et al. [76] demonstrated significant reversal of hypoxia and reversal of cytokine storm in patients hospitalized with severe COVID-19 following a single intravenous injection of bone marrow-derived exosomes (ExoFlo), with no adverse effects associated with the treatment. Although the number of COVID-19 patients who underwent MSC or MSC-Exo treatment is very limited and there is a lack of studies elucidating the underlying mechanisms, these studies showed the potential application of MSC therapy for severe COVID-19 cases. It is worth noted that risks associated with MSC transfusion appear to be uncommon. However, the potential risks might include failure of the cells to work as expected, potential for MSCs to multiply or change into inappropriate cell types, product contamination, growth of tumors, infections, thrombus formation, and administration site reactions [77, 78]. Fortunately, MSC-Exo can overcome these potential risks as they cannot replicate or differentiate [79].

Doctors and researchers from the UAE, USA, Iran, and Jordan are working on stem cell-based therapies for COVID-19, and these ongoing clinical trials are providing important information for the fight against COVID-19. Many studies have shown that MSCs-Exo treat ARDS by suppressing inflammation via modulating the immune systems and thus protect alveolar epithelial cells [54]. MSCs have also shown a positive impact against COVID-19-induced infection (ClinicalTrials.gov, Identifier: NCT04361942); they were found to be efficacious in reducing nonproductive inflammation and in promoting lung regeneration in phase 2 clinical trials (ClinicalTrials.gov, Identifier: NCT03608592), as well as in patients with ARDS in other clinical trials. The clinical trials are listed in Table 1, which summarizes the ongoing clinical trials, their country of origin, their current phase, and the possible working timeline. As shown in the table, some studies are still in the first phase, while others are in their second and third phases.

The recovery of patients is also documented through ClinicalTrials.gov, Identifier NCT04366063. The mortality rate in SARS-CoV-2-related severe ARDS is high despite treatment with antivirals, glucocorticoids, immunoglobulins, and mechanical ventilation. Preclinical and clinical evidence has indicated that MSCs migrate to the lungs and respond to the proinflammatory lung environment by releasing anti-inflammatory factors. Thus, they reduce the proliferation of proinflammatory cytokines while modulating regulatory T cells and macrophages to promote the resolution of inflammation [58]. Therefore, MSCs may have the potential to increase patients' survival by managing COVID-19 induced ARDS.

The primary objective of the phase-three trial is to appraise the potency and protection of the combination of the MSC remestemcel-L and standard of care compared to the placebo and standard of care in patients with ARDS due to SARS-CoV-2. Additionally, the trial is aimed at monitoring the effect of MSCs on inflammatory biomarkers (ClinicalTrials.gov Identifier: NCT04371393). Earlier research showed that MSCs could significantly reduce inflammatory cell infiltration in lung tissue and prevent lung tissue damage [58, 65], and previous trials (IRCT20200217046526N1) reported the safety of three injections of MSCs in patients infected with COVID-19. The clinical trials also show that MSC administration strongly improves the anti-inflammatory reactions in the body in COVID-19 patients [80]. A recent study (ClinicalTrials.gov, Identifier: NCT04346368) also mentioned the immunomodulatory effect of intravenously administered bone marrow MSCs, which modulated the lung microenvironment in COVID-19 patients. Critically ill COVID-19 patients with ARDS (mild or moderate) will be enrolled in this clinical trial. This multicenter trial will have 60 patients, and all patients in all groups will receive conventional therapy for virus treatment and supportive care for ARDS.

## 5. Summary and Conclusions

Wuhan has reported multiple cases of pneumonia patients infected with the novel coronavirus since December 2019. As of May 22, 2020, 5,223,401 cases of COVID-19 (per the applied case definitions and testing strategies in the affected countries) and 335,205 deaths have been reported (source: https://www.worldometers.com). In the future, the total number of cases in the world may exceed 10 million, and deaths may be around 3.4% of the total cases in most countries. Based on current data, 10–15% of the affected individuals will develop a severe form of the disease, requiring hospitalization and respiratory support. Thus, the COVID-19 pandemic is a public health emergency.

At present, no effective therapeutic strategy against COVID-19-induced pneumonia exists, especially for the severe and critical cases, and immediate therapy with minimal side effects is needed to overcome the crisis. Preclinical and clinical data support the investigational use of MSCs-Exo/EVs because of their anti-inflammatory and immunomodulatory responses. Moreover, lack of ethical restrictions, high availability, and easy isolation procedures are the key benefits of using MSCs-Exo-based therapy.

Intravenously infused stem cells provide a reliable alternative in terms of accumulation on the targeted site, multiplication, and differentiation due to paracrine activity. As the lungs are the primary organ infected by COVID-19, intravenous administration of MSCs-Exo may target the lungs to rescue the infected site from severe injury by providing immunomodulatory effects. Moreover, the noninvasive and economical qualities of MSCs make them an attractive therapeutic candidate for COVID-19.

The purpose of this review was to investigate the efficiency and safety of MSCs, especially MSCs-Exo, in treating patients with severe pneumonia infected with SARS-CoV-2. As recently recommended, MSCs should also be clinically validated for treating severe cases of MERS, for which mortality rates are up to 34% [74, 81]. Based on the clinical trials on COVID-19, this review encourages further research into the use of MSCs and/or EVs for treating COVID-19 after strictly following appropriate manufacturing procedures, quality control measurements, preclinical safety and efficacy data, and proper ethical regulations.

## Figures and Tables

**Table 1:** The available clinical trials (https://clinicaltrials.gov/) using MSCs and/or MSCs-Exo to treat COVID-19 patients (last accessed on July 21, 2020).

Status	Study title	Interventions	Country	Registered no.
Recruiting	Treatment of COVID-19 patients using Wharton's jelly-mesenchymal stem cells	WJ-MSCs	Jordan	NCT04313322
Completed	Study evaluating the safety and efficacy of autologous non-hematopoietic peripheral blood stem cells in COVID-19	Autologous nonhematopoietic peripheral blood stem cells (NHPBSC)	United Arab Emirates	NCT04473170
Not yet recruiting	Autologous adipose-derived stem cells (AdMSCs) for COVID-19	Autologous adipose-derived stem cells	United States	NCT04428801
Recruiting	Mesenchymal stem cell infusion for COVID-19 infection	Mesenchymal stem cells	Pakistan	NCT04444271
Recruiting	Safety and efficacy study of allogeneic human dental pulp mesenchymal stem cells to treat severe COVID-19 patients	Allogeneic human dental pulp stem cells (BSH BTC & Utooth BTC)	China	NCT04336254
Not yet recruiting	Safety and efficacy of mesenchymal stem cells in the management of severe COVID-19 pneumonia	Umbilical cord-derived mesenchymal stem cells	Spain	NCT04429763
Recruiting	MSCs in COVID-19 ARDS	Mesenchymal stromal cells	United States	NCT04371393
Recruiting	Human umbilical cord mesenchymal stem cells (MSCs) therapy in ARDS (ARDS)	Umbilical cord-derived mesenchymal stem cell (UCMSCs) suspension	China	NCT03608592
Recruiting	Mesenchymal stem cell for acute respiratory distress syndrome due for COVID-19	Infusion IV of mesenchymal stem cells	Mexico	NCT04416139
Not yet recruiting	NestaCell® mesenchymal stem cell to treat patients with severe COVID-19 pneumonia	NestaCell®	Brazil	NCT04315987
Enrolling by invitation	A randomized, double-blind, placebo-controlled clinical trial to determine the safety and efficacy of Hope Biosciences Allogeneic Mesenchymal Stem Cell Therapy (HB-adMSCs) to provide protection against COVID-19	HB-adMSCs	United States	NCT04348435
Active, not recruiting	Use of mesenchymal stem cells in acute respiratory distress syndrome caused by COVID-19	Mesenchymal stem cells derived from Wharton jelly of umbilical cords	Mexico	NCT04456361
Recruiting	Clinical trial to assess the safety and efficacy of intravenous administration of allogeneic adult mesenchymal stem cells of expanded adipose tissue in patients with severe pneumonia due to COVID-19	Allogeneic and expanded adipose tissue-derived mesenchymal stem cells	Spain	NCT04366323
Enrolling by invitation	A clinical trial to determine the safety and efficacy of Hope Biosciences Autologous Mesenchymal Stem Cell Therapy (HB-adMSCs) to provide protection against COVID-19	HB-adMSCs	United States	NCT04349631
Not yet recruiting	Novel coronavirus induced severe pneumonia treated by dental pulp mesenchymal stem cells	Dental pulp mesenchymal stem cells	China	NCT04302519
Recruiting	Mesenchymal stem cell treatment for pneumonia patients infected with COVID-19	MSCs	China	NCT04252118
Not yet recruiting	Bone marrow-derived mesenchymal stem cell treatment for severe patients with coronavirus disease 2019 (COVID-19)	BM-MSCs	China	NCT04346368
Active, not recruiting	Treatment with human umbilical cord-derived mesenchymal stem cells for severe corona virus disease 2019 (COVID-19)	UC-MSCs	China	NCT04288102
Not yet recruiting	Study of human umbilical cord mesenchymal stem cells in the treatment of severe COVID-19	UC-MSCs	China	NCT04273646
Recruiting	Efficacy of intravenous infusions of stem cells in the treatment of COVID-19 patients	Intravenous infusions of stem cells	Pakistan	NCT04437823
Enrolling by invitation	Treatment of Covid-19 associated pneumonia with allogenic pooled olfactory mucosa-derived Mesenchymal stem cells	Allogenic pooled olfactory mucosa-derivedmesenchymal stem cells	Belarus	NCT04382547
Recruiting	Clinical research of human mesenchymal stem cells in the treatment of COVID-19 pneumonia	UC-MSCs	China	NCT04339660
Active, not recruiting	Safety and effectiveness of mesenchymal stem cells in the treatment of pneumonia of coronavirus disease 2019	Mesenchymal stem cells	China	NCT04371601
Recruiting	Mesenchymal stem cell therapy for SARS-CoV-2-related acute respiratory distress syndrome	Cell therapy protocol 1 Cell therapy protocol 2	Iran	NCT04366063
Active, not recruiting	Role of immune and inflammatory response in recipients of allogeneic haematopoietic stem cell transplantation (SCT) affected by severe COVID19		United Kingdom	NCT04349540
Recruiting	Administration of allogenic UC-MSCs as adjuvant therapy for critically-ill COVID-19 patients	Umbilical cord mesenchymal stem cells	Indonesia	NCT04457609
Recruiting	Use of UC-MSCs for COVID-19 patients	Umbilical cord mesenchymal stem cells	United States	NCT04355728
Recruiting	Efficacy and safety study of allogeneic HB-adMSCs for the treatment of COVID-19	HB-adMSC	United States	NCT04362189
Recruiting	Clinical use of stem cells for the treatment of Covid-19	MSC treatment	Turkey	NCT04392778
Not yet recruiting	Stem cell educator therapy treat the viral inflammation in COVID-19	Combination product: stem cell educator-treated mononuclear cells apheresis	United States	NCT04299152
Recruiting	Treatment of coronavirus COVID-19 pneumonia (pathogen SARS-CoV-2) with cryopreserved allogeneic P_MMSCs and UC-MMSCs	Placenta-derived MMSCs; cryopreserved placenta-derived multipotent mesenchymal stromal cells	Ukraine	NCT04461925
Not yet recruiting	BAttLe against COVID-19 using mesenchYmal stromal cells	Allogeneic and expanded adipose tissue-derived mesenchymal stromal cells	United States	NCT04348461
Not yet recruiting	Safety and efficacy of intravenous Wharton's jelly derived mesenchymal stem cells in acute respiratory distress syndrome due to COVID 19	Wharton's jelly-derived mesenchymal stem cells	Colombia	NCT04390152
Not yet recruiting	Use of hUC-MSC product (BX-U001) for the treatment of COVID-19 with ARDS	Human umbilical cord mesenchymal stem cells	United States	NCT04452097
Recruiting	Pediatrics HOT COVID-19 database in NY tristate		United States	NCT04445402
Not yet recruiting	Using PRP and cord blood in treatment of Covid -19	Stem cells	Egypt	NCT04393415
Not yet recruiting	Study of the safety of therapeutic Tx with Immunomodulatory MSC in adults with COVID-19 infection requiring mechanical ventilation	BM-Allo.MSC	United States	NCT04397796
Recruiting	Safety and efficacy of CAStem for severe COVID-19 associated with/without ARDS	CAStem	China	NCT04331613
Recruiting	Mesenchymal stromal cell therapy for the treatment of acute respiratory distress syndrome	Mesenchymal stromal stem cells—KI-MSC-PL-205	Sweden	NCT04447833
Not yet recruiting	Mesenchymal stem cells (MSCs) in inflammation-resolution programs of coronavirus disease 2019 (COVID-19) induced acute respiratory distress syndrome (ARDS)	MSC	Germany	NCT04377334
Recruiting	Efficacy and safety evaluation of mesenchymal stem cells for the treatment of patients with respiratory distress due to COVID-19	XCEL-UMC-BETA	Spain	NCT04390139
Not yet recruiting	Cellular immuno-therapy for COVID-19 acute respiratory distress syndrome—vanguard	Mesenchymal stromal cells	Canada	NCT04400032
Not yet recruiting	ACT-20 in patients with severe COVID-19 pneumonia	ACT-20-MSC	United States	NCT04398303
Not yet recruiting	Safety and feasibility of allogenic MSC in the treatment of COVID-19	Mesenchymal stromal cell infusion	Brazil	NCT04467047
Recruiting	Repair of acute respiratory distress syndrome by stromal cell administration (REALIST) (COVID-19)	Human umbilical cord-derived CD362 enriched MSCs	United Kingdom	NCT03042143
Not yet recruiting	Mesenchymal stromal cells for the treatment of SARS-CoV-2 induced acute respiratory failure (COVID-19 disease)	Mesenchymal stromal cells	United States	NCT04345601
Recruiting	Treatment of severe COVID-19 pneumonia with allogeneic mesenchymal stromal cells (COVID_MSV)	Mesenchymal stromal cells	Spain	NCT04361942
Recruiting	Double-blind, multicenter, study to evaluate the efficacy of PLX PAD for the treatment of COVID-19	PLX-PAD	United States	NCT04389450
Recruiting	Umbilical cord(UC)-derived Mesenchymal stem cells(MSCs) treatment for the 2019-novel coronavirus (nCOV) pneumonia	UC-MSCs	China	NCT04269525
Recruiting	Cell therapy using umbilical cord-derived mesenchymal stromal cells in SARS-CoV-2-related ARDS	Umbilical cord Wharton's jelly-derived human	France	NCT04333368
Recruiting	MultiStem administration for COVID-19 induced ARDS (MACoVIA)	MultiStem	United States	NCT04367077
Not yet recruiting	Multiple dosing of mesenchymal stromal cells in patients with ARDS (COVID-19)	Mesenchymal stromal cells	United States	NCT04466098
Not yet recruiting	A pilot clinical study on inhalation of mesenchymal stem cells exosomes treating severe novel coronavirus pneumonia	MSCs-derived exosomes	China	NCT04276987
Not yet recruiting	A study to collect bone marrow for process development and production of BM-MSC to treat severe COVID19 pneumonitis	Bone marrow harvest	United Kingdom	NCT04397471
Not yet recruiting	Organicell flow for patients with COVID-19	Organicell flow (human amniotic fluid contain growth factors, cytokines, and chemokines as well as other extracellular vesicles/nanoparticles derived from amniotic stem and epithelial cells	United States	NCT04384445

## Data Availability

All data are available in the manuscript.

## Supplementary Materials

Supplementary Materials Video. 1: this video show the possible mechanism of MSCs-Exo for treating COVID-19 symptoms.
